# Blockade of the Adenosine A_3_ Receptor Attenuates Caspase 1 Activation in Renal Tubule Epithelial Cells and Decreases Interleukins IL-1β and IL-18 in Diabetic Rats

**DOI:** 10.3390/ijms20184531

**Published:** 2019-09-13

**Authors:** Wallys Garrido, Claudia Jara, Angelo Torres, Raibel Suarez, Claudio Cappelli, Carlos Oyarzún, Claudia Quezada, Rody San Martín

**Affiliations:** Institute of Biochemistry and Microbiology, Science Faculty, Universidad Austral de Chile, Valdivia 5110566, Chile; wallys.garrido@uach.cl (W.G.); claudia.jaracancino@gmail.com (C.J.); postdoctorado3180749@gmail.com (A.T.); raibelsua@gmail.com (R.S.); claudio.cappellileon@gmail.com (C.C.); carlosoyarzun@uach.cl (C.O.); claudiaquezada@uach.cl (C.Q.)

**Keywords:** adenosine receptor, diabetic nephropathy, interleukins 1 and 18, renal fibrosis, inflammasome

## Abstract

Diabetic nephropathy (DN) is the main cause of end-stage renal disease, which remains incurable. The progression of DN is associated with progressive and irreversible renal fibrosis and also high levels of adenosine. Our aim was to evaluate the effects of ADORA3 antagonism on renal injury in streptozotocin-induced diabetic rats. An ADORA3 antagonist that was administered in diabetic rats greatly inhibited the levels of inflammatory interleukins IL-1β and IL-18, meanwhile when adenosine deaminase was administered, there was a non-selective attenuation of the inflammatory mediators IL-1β, IL-18, IL-6, and induction of IL-10. The ADORA3 antagonist attenuated the high glucose-induced activation of caspase 1 in HK2 cells in vitro. Additionally, ADORA3 antagonisms blocked the increase in caspase 1 and the nuclear localization of NFκB in the renal tubular epithelium of diabetic rats, both events that are involved in regulating the production and activation of IL-1β and IL-18. The effects of the A3 receptor antagonist resulted in the attenuation of kidney injury, as evidenced by decreased levels of the pro-fibrotic marker α-SMA at histological levels and the restoration of proteinuria in diabetic rats. We conclude that ADORA3 antagonism represents a potential therapeutic target that mechanistically works through the selective blockade of the NLRP3 inflammasome.

## 1. Introduction

Diabetes mellitus is a metabolic disease with a high prevalence worldwide and it is considered to be one of the main causes of morbidity and mortality [1,2]. One of the complications of diabetes is diabetic nephropathy (DN), which affects 30–40% of all diabetics, positioning itself as the first cause of end-stage renal disease and also multiplies cardiovascular risk [3]. To date, there is no cure for DN, and patient care aims to reduce the most important risk factors, such as poor glycaemic control and arterial hypertension [3,4]. The use of blockers of the renin angiotensin system have renoprotective effects, although their efficacy in patients is limited to only slowing the progression to End-Stage Renal Disease (ESRD) [5,6]. Importantly, the progression of the DN generates high costs to health systems associated with the care of patients because it predisposes to the use of organ replacement therapies that include haemodialysis and kidney transplantation [1,7].

In early stages, DN is characterized by podocytopathy and alterations to the filtration barrier, as clinically evidenced by hyperfiltration and microalbuminuria [8,9,10]. The evolution of this disease is associated to progressive and irreversible renal fibrosis [11], which is orchestrated by infiltrating cells and the acquisition of profibrotic phenotypes of renal resident cells, which leads to loss of functionality and renal regenerative capacity [12,13,14,15]. Part of this process is triggered, because renal cells, such as tubular epithelial cells and mesangial cells [16], subjected to an insult, at least metabolic and ischemic in DN, trigger an inflammatory process and secret a series of pro-inflammatory cytokines and chemokines [17,18]. These signaling molecules induce the infiltration of immune cells into glomeruli and interstitium [19] and the persistence of signals that directly or indirectly contribute to the accumulation of interstitial myofibroblasts that expresses α-SMA, type II intermediate filaments (desmin and vimentin), and secrete proteins and ECM [20,21,22,23,24]. Currently, the identification of mediators of this fibrotic process and the evaluation of possible therapeutic targets is of great clinical relevance.

A few years ago, the progression of DN in humans and renal fibrosis in experimental models was correlated with the increased production of the extracellular adenosine nucleoside, while diabetic patients without renal complications had basal levels of this nucleoside, like those that wer found in healthy individuals [25,26]. Extracellular adenosine arises from the metabolism of precursor adenine nucleotides due to the activity of a series of ectonucleotidases and by variations in the activity of nucleoside transporters [27]. Adenosine mediates its function through the activation of a family of four purinergic receptors coupled to G protein, which include the adenosine A1, A2A, A2B, and A3 receptors [28]. Recent studies using models of kidney injury have associated the adenosine A3 receptor (ADORA3) with the progression of cellular events conducting to renal fibrosis, which can be reversed after the use of a receptor antagonist [29,30], which would be particularly relevant when the ligand levels are increased, as is the case of DN [27,31]. However, the mechanism by which adenosine and signalling through its A3 receptor could mediate fibrosis is still unknown. 

There are some inflammatory cytokines that appear to play a key role in the progression of renal damage during DN, among these IL-1β (Interleukin 1β), IL-6, IL-18, and TNF-α [32,33,34,35]. High levels of these cytokines have been found in the urine of patients with DN, which has been related to increased albuminuria [36]. IL-1β and IL-18 are synthesized as inactive cytoplasmic precursors (pro-IL1β and pro IL18), and their maturation depends on the activity of the enzyme caspase 1 [36]. Interestingly, this enzyme is overexpressed in the kidneys of diabetic rats [35,37]. The profibrotic effects of these cytokines are mediated by the intracellular activity of NF-κB (nuclear factor enhancer of the light chains kappa of activated B cells), which is a transcription factor whose function is also associated with the progression of DN [36]. 

The antagonists of the A3 subtype of adenosine receptors have anti-inflammatory properties [38], therefore our aim was to evaluate the inhibitory potential of an ADORA3 antagonist against renal fibrosis and its association with the generation of inflammatory mediators, such as those that are derived from the NLP3 inflammasoma.

## 2. Results

### 2.1. Adenosine Deaminase Administered in Vivo Affects the Urinary Profile of Cytokines in Diabetic Rats

**Figure 1:**
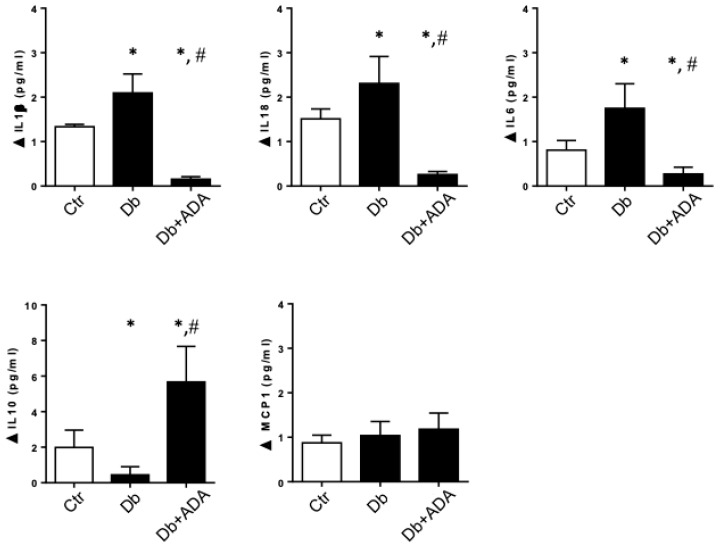
The administration of adenosine deaminase in diabetic rats affects the levels of cytokines. The pegylated-adenosine deaminase (ADA) or vehicle were administered to diabetic rats for a four weeks period. The levels of the cytokines IL-1β, IL-18, IL-6, IL-10, and MCP-1 were quantified in urines of rats by using Immunoassays & MILLIPLEX^®^ map system. The control group (Ctr) corresponded to non-diabetic rats (*n* = 6), the diabetic group (Db) corresponded to streptozotocin (STZ)-induced diabetic rats treated with vehicle (*n* = 5) and the diabetic treated group (Db + ADA) corresponded to STZ-induced diabetic rats that received ADA at doses 5U/Kg per week (*n* = 6). *, *p* < 0.05 vs. control; ^#^, *p* < 0.05 vs. Diabetic.

The increase in extracellular adenosine has been correlated with the progression of DN, but the mechanisms of pathogenicity have not been fully characterized. Therefore, the use of the enzyme adenosine deaminase (ADA), which converts adenosine to inosine and thereby ends its biological effects through its receptors, is a tool that allows us to explore these mechanisms. Thus, treatment of diabetic rats with ADA attenuated proteinuria and decreased the levels of the α-SMA fibrosis marker in renal tissue [39]. In this study, we determined the effect of ADA treatment on pro-inflammatory cytokine levels in the urine of diabetic rats. One month after the induction of experimental diabetes using STZ, the rats were treated with ADA or vehicle for four weeks. In the urine of diabetic rats that were treated with ADA, a significant decrease in the amount of the inflammatory cytokines IL-1β, IL-6, and IL-18 (Figure 1) were observed when compared to diabetic rats that were treated with vehicle. The levels of these cytokines in the ADA-treated rats were even lower than the basal levels of the control rats. On the contrary, the level of the anti-inflammatory cytokine IL-10 increased in diabetic rats after treatment with ADA. Regarding the levels of MCP-1, no significant changes were observed in our model in diabetic rats or after treatment with ADA (Figure 1). Thus, we conclude that adenosine and signalling through its receptors may be implicated in the proinflammatory milieu that contributes to renal injury in the diabetic kidney disease.

### 2.2. The ADORA3 Antagonism Decreases the Urine Content of IL-1β and IL18 in Diabetic Rats 

It was previously demonstrated that the use of ADORA3 antagonists have anti-inflammatory effects [38], however the renoprotective properties of the use of these antagonists in diabetic nephropathy has been suggested, but not mechanistically understood [40]. Our first approach was to evaluate the physiological effects of the ADORA3 antagonists administered to rats following one month from induction of experimental diabetes for a period of four weeks. The antagonists that were utilized for treatments were MRS1220 highly selective for human ADORA3, but with lower affinity for this receptor in rats, therefore the studies in animals were further corroborated while using MRS1523. As shown in Table 1, the diabetic rats had an impeded gain of body weight, elevated blood glucose levels, and increased kidney weight with respect to total body weight as compared to the control non-diabetic rats. Non-significant differences in these parameters were observed after treatment with ADORA3 antagonists. Meanwhile, proteinuria decreased in diabetic rats treated with the ADORA3 antagonist as compared to diabetic rats that were injected with vehicle.

**Figure 2:**
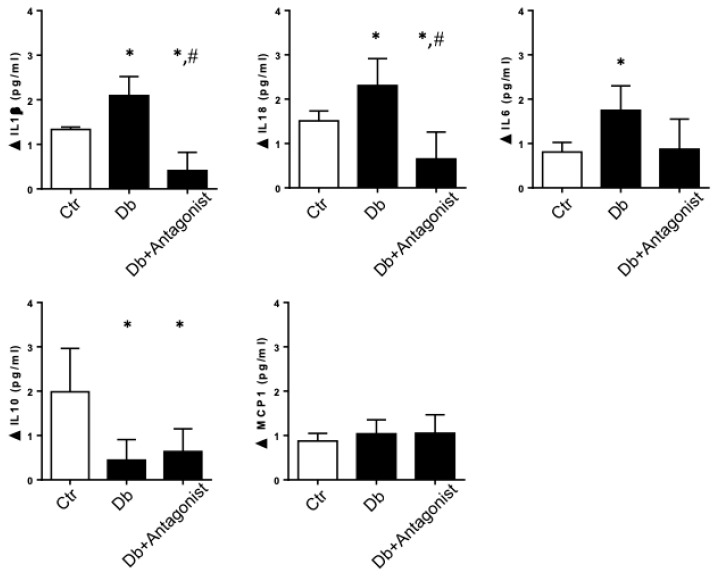
The administration of an antagonist of the ADORA3 decreases IL-1β and IL-18 in diabetic rats. ADORA3 antagonist or vehicle were administered to diabetic rats for a four weeks period. The levels of the cytokines IL-1β, IL-18, IL-6, IL-10, and MCP-1 were quantified in urines of rats by using Immunoassays & MILLIPLEX^®^ map system. The control group (Ctr) corresponded to non-diabetic rats (*n* = 5), the diabetic group (Db) corresponded to STZ-induced diabetic rats treated with vehicle (*n* = 5) and the diabetic treated group (Db + Antagonist) corresponded to STZ-induced diabetic rats that received ADORA3 antagonist (*n* = 5). *, *p* < 0.05 vs. control; ^#^, *p* < 0.05 vs. Diabetic.

When evaluating urinary secretion of inflammatory cytokines in rats with diabetes, the use of the antagonist greatly decreased the levels of the cytokines IL-1β and IL18, which were increased by diabetes (Figure 2). Unlike what happened when treating diabetic rats with ADA, ADORA3 antagonism does not significantly affect the levels of IL-10, while only attenuates the levels of IL-6 found in diabetes (Figure 2).

### 2.3. The ADORA3 Antagonism Affects Selectively Caspase 1 Activation

**Figure 3:**
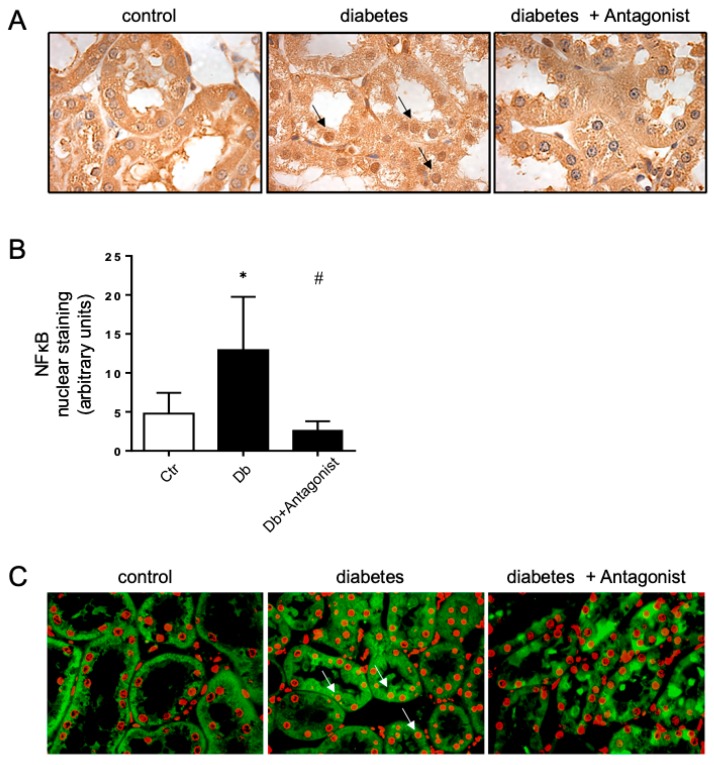
The ADORA3 antagonist decreases nuclear localization of NF-κB. Analyses of the subcellular distribution of NF-κB in the kidney cortex were done in renal samples from non-diabetic control rats (Ctr), STZ-induced diabetic rats (Db) and STZ-induced diabetic rats that were treated with ADORA3 antagonist (Db + Antagonist). Representative images of localization of NF-κB in tubule epithelial cells obtained by peroxidase-coupled immunostaining are shown in (**A**). Arrows indicate positive nuclear staining to NF-κB. The graph in (**B)** depicts the quantitative analysis of nuclear staining of NF-κB on 10 microscopic fields per slice from animals belonging to each experimental group using UN-SCANIT 2.0 software. *n* = 5 in each group. *, *p* < 0.05 vs. control group; ^#^, *p* < 0.05 vs. Diabetic group. Representative merged images of NF-κB immunofluorescence (green) and nuclei staining using DAPI (red) in each experimental group are shown in (**C**). Arrows indicate representative colocalization of NF-κB and nuclear staining. Original magnification 400×.

During kidney damage, NF-κB has a prominent role in the inflammatory response. Under diabetic conditions, NF-κB is activated and it translocates to the nucleus, joining to the promoter region of several genes that were considered to be significant in the progression DN. Under normal conditions, NF-κB is latent and inactive in the cytoplasm. For this reason, we evaluated the effect of the ADORA3 antagonism on the subcellular distribution of NF-κB in renal cells. While using immunohistochemistry, we detected that the nuclear distribution of NF-κB was increased in the tubular epithelial cells of the kidney in diabetic rats (Figure 3A). However, the treatment of diabetic rats with an ADORA3 antagonist decreased their nuclear distribution (Figure 3B). This effect was also evident when evaluating the colocalization of NF-κB with the nuclear DAPI staining, with a loss of colocalization in the diabetic rats treated with antagonist as compared diabetic rats without treatment (Figure 3C).

**Figure 4:**
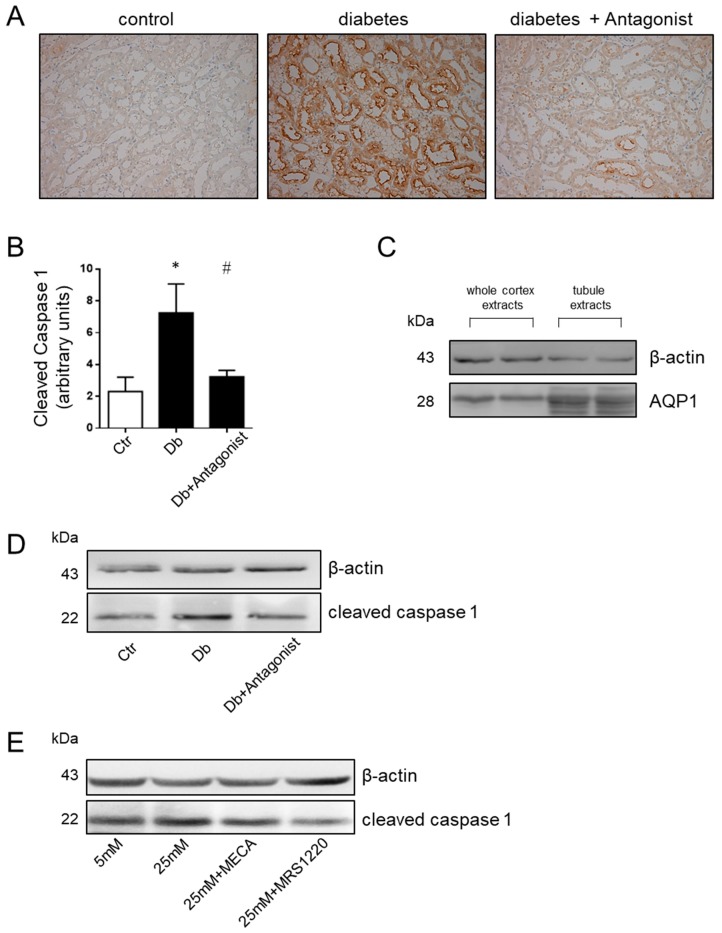
The ADORA3 antagonist reverses caspase 1 activation in diabetic rats in vivo and in cells *in vitro*. The levels of cleaved caspase 1 in the kidney sections from experimental group were assayed by immunohistochemistry. Representative images of cleaved caspase 1 distribution in kidney cortex are shown in (**A**) using MRS1220 as antagonist. Original magnification 400×. The graph in (**B**) depicts the quantitative analysis of caspase 1 staining on 10 microscopic fields per slice from animals belonging to each experimental group using the UN-SCANIT 2.0 software. *n* = 5 in each group. *, *p* < 0.05 vs. control group; ^#^, *p* < 0.05 vs. Diabetic group. In (**C**) is shown the enrichment of aquaporin1 (AQP1), a marker of proximal tubules, in the fraction of tubules purified from kidney cortex. The western blot shown in (**D**) indicates the reversion of the increases of cleaved caspase 1 in the proximal tubules-enriched fraction from diabetic rats treated with the antagonist of ADORA3. β-actin was used as loading control. The in vitro effects of ADORA3 modulation in HK2 cells is shown in (**E**). The western blot shows the attenuation of activated caspase 1 by the ADORA3 antagonist MRS1220 in HK2 cells exposed to high d-glucose concentrations of 25 mM.

**Figure 5:**
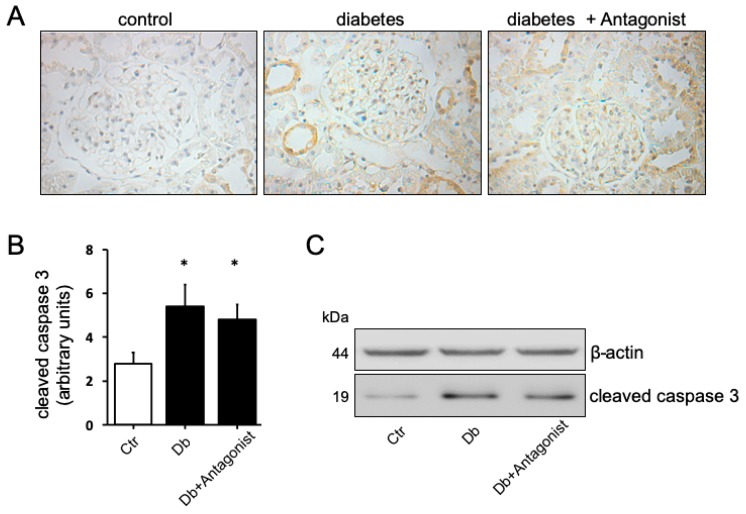
The ADORA3 antagonism does not affects caspase 3 activation in the kidney of diabetic rats. The levels of cleaved caspase 3 in the kidney sections from experimental groups were assayed by immunohistochemistry. Representative images of cleaved caspase 3 distribution in kidney cortex are shown in (**A**). Original magnification 400×. The graph in (**B**) depicts the quantitative analysis of caspase 3 staining on 10 microscopic fields per slice from animals belonging to each experimental group using the UN-SCANIT 2.0 software. *n* = 5 in each group. *, *p* < 0.05 vs. control group. In (**C**) is shown the western blot analysis of cleaved caspase 3 in total kidney protein extracts from experimental groups. β-actin was used as the loading control.

Additionally, a relevant aspect that is associated with the production of IL-1β and IL18 is caspase 1 activity. While using immunohistochemical analysis, we observed that diabetic rats present an increase in the content of the active, proteolyzed at Asp296, form of caspase 1 compared to the basal content present in control rats (Figure 4A). The pattern of caspase 1 distribution was corresponding with the distribution of aquaporin 1 in proximal tubule epithelial cells [31]. The use of the ADORA3 antagonists MRS1220 or MRS1523 blocked the diabetes-inducing effect on this form of the protease (Figure 4B). While using protein extracts of a purified proximal tubules-enriched fraction from the study groups, we found that the content of the active form of caspase 1 is higher in diabetic rats and that treatment with the ADORA3 antagonists reversed this increase (Figure 4C,D). Human proximal tubule epithelial cells line HK2 was exposed to physiological or high d-glucose condition for 24 h to further confirm the role of ADORA3 on activation of caspase 1. This treatment increased the levels of cleaved caspase 1, but the use of MRS1220 reversed this effect (Figure 4E). Additionally, we analysed the activation of apoptotic caspase 3 in the kidney of diabetic rats, finding increased levels in diverse types of epithelial tubule cells when compared to control rats (Figure 5). Different to the situation that was observed with caspase 1, the activation of caspase 3 was seem non affected by the treatment with the antagonist of ADORA3 when immunohistochemically evaluated in tissue sections or by western blots in total kidney protein extracts (Figure 5).

### 2.4. The Antagonist of the ADORA3 Attenuates Renal Injury in Diabetic Rats

**Figure 6:**
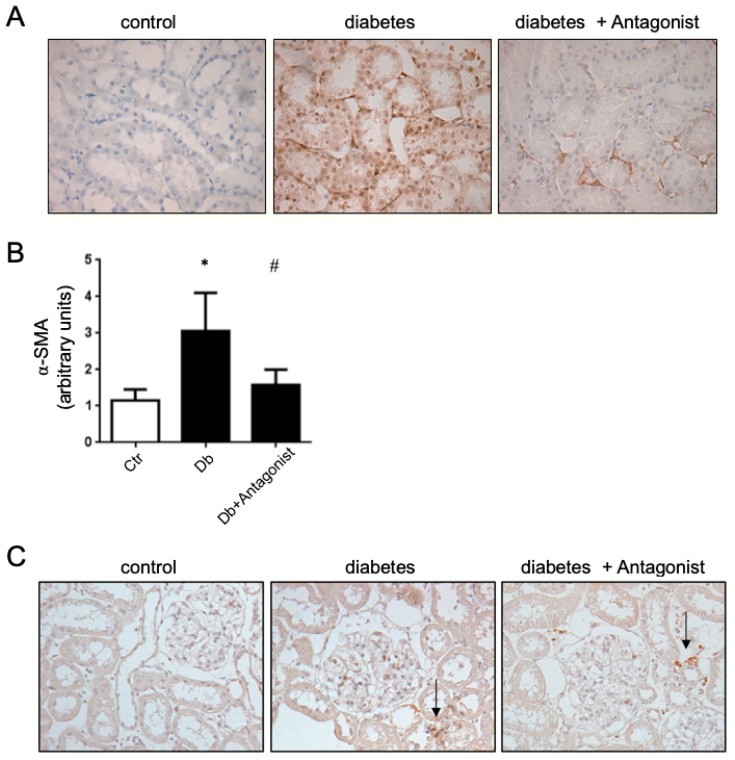
The antagonist of ADORA3 attenuates profibrotic activation of cells in diabetic rats. (**A**) The images are representatives of immunohistochemical detection of α-SMA in kidney sections from experimental groups. Original magnification 400×. (**B**) The graph shows the quantitative analysis of α-SMA staining on 10 microscopic fields per slice from animals belonging to each experimental group using the UN-SCANIT 2.0 software. *n* = 5 in each group. *, *p* < 0.05 vs. control group; ^#^, *p* < 0.05 vs. Diabetic group. (**C**) The images are representatives of tubulointerstitial infiltrating monocyte/macrophages CD68+ in the kidney cortex of experimental groups. Arrows indicates infiltrating CD68+ cells. Original magnification 400×.

By immunohistochemistry, we evidenced that the ADORA3 has a wide distribution in renal tubular epithelium, and further the diabetic condition in rats did not alter the contents of this receptor in the kidney [29]. However, beyond ADORA3 antagonists downregulation IL-1β and IL18, it had consequences on the progression of renal injury in the diabetic kidney. Functionally, the ADORA3 antagonism attenuated the proteinuria in treated rats (Table 1). Furthermore, the ADORA3 antagonism has the potential to block renal fibrosis in diabetic rats, since the distribution of the pro-fibrotic marker α-SMA was markedly decreased at the renal level, mainly at the tubulointertitium (Figure 6A,B). This effect could not be attributed to a reduction in monocyte/macrophage cells infiltrating the tubulointertitium, because the antagonism does not greatly influence the arrival of CD68 positive cells in the kidney cortex in our model (Figure 6C).

## 3. Discussion

In this study, we demonstrated that treatment with an ADORA3 antagonist provides a renoprotective effect during the early stages of kidney damage in STZ-induced diabetic rats by decreasing the proinflammatory cytokines IL-1β and IL18. Recently, it was shown that renal alterations in the db/db mice were similarly prevented by using a novel ADORA3 antagonist [40], therefore our work contributes to the identification of the renoprotective mechanism mediated by the ADORA3 antagonists. The model of experimental diabetes in rats using STZ allows for early events associated with kidney damage to be studied, among them the loss of adenosine homeostasis has been established with the progression of DN [27]. In fact, the increase of adenosine by itself can generate renal alterations that resemble those that are found in DN, such as proteinuria and renal fibrosis [34]. Interestingly, the increase in extracellular adenosine can result from the metabolic and hormonal breakdown present in diabetes, such as the deficient activity of insulin, and directly affect the uptake and extracellular metabolism of adenosine at the renal level [39]. 

We concluded that A3 receptor signaling, in part, could mediate the increased production of IL1 and IL18 in the renal cells. Previously, it has been established that mononuclear phagocytes and the tubular epithelial cells in the kidney possess all of the components that are necessary to produce these interleukins, while there are still controversies about the ability of glomerular cells to produce them [36,41]. Although the present study did not regarding preciselly the cellular origin of these interleukins, the distribution of the ADORA3 mainly in tubule epithelial cells [27,29] and its correlation with the increase in caspase 1 indicates that the renal tubular epithelium might be the main source. In addition, there is no significant infiltration of inflammatory cells in our experimental model, where early alterations in renal pathology are developed, so it is presumed that their contribution would be less relevant. Both interleukins are produced as inactive precursors, with caspase 1 being the enzyme that is responsible for their maturation. Recent studies indicate that caspase 1 is overexpressed in diabetic rat kidneys and in mesangial cells exposed to high concentrations of glucose [42,43], which corresponds to our results where we observed higher activated caspase 1 content in the renal cortex of diabetic rats. Further, some authors [44] reported that intrarenal NLRP3/Caspase 1 inflammasome activation was present in both the db/db mouse and the murine STZ-induced type 1 diabetes model. Additionally, the lack of caspase 1 protected against the development of nephropathy in STZ-induced diabetic animals. At present, no previous studies have related the levels of this enzyme with adenosine signaling in diabetic nephropathy, but it is suggested that the use of the ADORA3 antagonist could have a major role on caspase 1 activation, which is a necessary event in the generation of interleukins. We do not know whether the adenosine receptor has a direct or indirect effect on the activation of caspase 1, however some studies describe the effect of adenosine receptors on the activation of the NLPR3/caspase 1 axis in certain cell types [45]. 

IL-1β and IL-18 appear to contribute to the progression of renal damage during diabetic nephropathy. Its production progressively increases in the kidney of diabetic animals and in the case of humans its levels correlate with the degree of albuminuria [37,46,47]. Further, IL-1R [48] and IL-18 [49] have been directly associated with the promotion of renal fibrosis. IL-1β, together with TGF-β1, are associated to the mesenchymal transition of renal cells, which contribute to the progression of renal damage [32], while mice that are deficient in IL-18 are protected from ischaemia reperfusion injury with fewer intrarenal neutrophils and macrophages, and less production of inflammatory mediators [50]. Interestingly, the profibrotic effects of these cytokines are mediated by intracellular signaling that involves NF-κB [33], which is a transcription factor whose function has been well recognized in the progression of DN [51]. Its activation mainly depends on the phosphorylation and degradation of IκB and subsequent translocation to the nucleus [36]. Activation of NF-κB is higher in the kidneys of diabetic animals and in the human disease [52,53]. Thus, our results demonstrated the association between the inhibition of NF-κB levels in the nucleus, lower levels of urinary IL1β and IL18, and the attenuation of the profibrotic α-SMA renal marker with the use of the ADORA3 antagonist in diabetic rats, evidencing, for the first time, a role for adenosinergic signaling in modulating NLRP3 inflammasome in the kidney. 

Some studies have described the expression and distribution of adenosine receptor subtypes and their correlation with the progression of DN [27,54]. The location of the ADORA3 receptor has been detected in glomerular, and mainly in tubular epithelial cells of the rat cortex, showing that their total levels do not significantly change between healthy and diabetic rats [27,29]. However, another study indicates that membrane-associated ADORA3 protein levels increased by 70% in diabetic kidney cortex, while it decreased by 80% in medulla [54], which may indicate that its signaling properties may be different in DN since classically this receptor is rapidly desensitized in the presence of the ligand [55]. It was previously determined that this receptor can mediate apoptosis in kidney cells [56,57]. Additionally, in in vitro studies, the use of an ADORA3 antagonist attenuates the expression of profibrotic activation markers α-SMA and fibronectin in response to TGF-β in HK2 cells [58]. Different to the observations in our model of early renal damage, in the biopsies of patients with DN who present advanced renal injury, an increase of the ADORA3 receptor in tubulointerstitial cells has been observed, which could be related to the proliferation and infiltration of cells that mediate the progression of interstitial fibrosis [29]. However, it has been also revealed that an early event that is associated with renal fibrosis involves mesenchymal-like phenotypic transdifferentiating of epithelial tubule cells, which orchestrate a profibrotic cascade through proinflammatory and profibrotic factors release and the secretion of extracellular matrix components [12]. Thus, while considering our results, we conclude that ADORA3 may have a role in this early event. 

Together, these results support the evaluation of ADORA3 receptor blockers as a new therapeutic strategy for the treatment of DN that is aimed at attenuating the profibrotic cascade that leads to progressive renal disease.

## 4. Materials and Methods

### 4.1. Materials

Adenosine A_3_ receptor antagonist MRS1220 was from Tocris Biosciences and MRS1523 from Santa Cruz Biotechnology. The chemical products, Immunoassays & MILLIPLEX^®^ map system and streptozotocin were purchased from Merck KGaA. The adenosine deaminase extracted from Bovine was obtained from Sigma.

### 4.2. Animals Models

Diabetes was induced in male rats (Sprague-Dawley) weighing 250 g by single intravenous administration of streptozotocin (STZ) at 55 mg/kg dissolved in citrate buffer, pH 4.5. The control rats were injected with an equivalent volume of vehicle. The diabetic groups included animals that present blood glucose levels ≥ 25 mmol/L.

### 4.3. Animal Treatments

Adenosine deaminase was PEGylated as previously described [34]. Following one month from diabetes induction in rats, adenosine deaminase (5 U/Kg) was administered weekly by intraperitoneal injection for a four-week period. Additionally, diabetic rats were treated with the adenosine A_3_ receptor antagonist MRS1220 at doses 0.1 mg/kg or MRS1523 at doses 0.1 mg/kg via intraperitoneal administration from days 31 to 60 post diabetes induction. Control diabetic rats were administered with an equivalent volume of the vehicle PBS 1X. 

### 4.4. Ethics Statement

All applicable international, national, and/or institutional guidelines for the care and use of animals were followed. All of the animal procedures in this study granted by FONDECYT 1171340 and 3150548 were approved by the Institutional Committee for the Use of Live Animals in Research at the University Austral de Chile (Ref. 59 of year 2012). Rats were euthanized by intraperitoneal injection of sodium thiopental (200 mg/kg body weight).

### 4.5. Quantification of Cytokines in Urine

Urine was collected to measure the inflammatory cytokines by Immunoassays & MILLIPLEX^®^ map system designing for interleukin IL-1β, IL-6, IL-10, IL-18, tumor necrosis factor (TNF) -α, and MCP1. Briefly, each layout of the test plate consisted of six standards in duplicate, two positive controls in duplicate, two blank wells, and up to 78 urine samples. At the time of the assay, the samples were thawed on ice and then centrifuged at 20,000× *g* for 10 min. at 4 °C, using the supernatant for analysis. The urine samples from the control and diabetic rats with or without treatment were analyzed in triplicate on the same plate. The samples and standards were processed while using the Luminex 100 IS instrument platform and the related Luminex 100 IS software (version 2.3, Luminex Corporation, Austin, TX, USA). The readings were analyzed with the standard version of the Millplex Analyst software (Merck KGaA, Darmstadt, Germany). A five-parameter logistic regression model was used to create the standard curves (pg/mL) and calculate the mean sample concentration of each triplicate.

### 4.6. Proteinuria

The urine of rats were recollected in a metabolic cage for 24 h and the protein contents were quantified while using the pyrogallol red molybdate method (Proti U/LCR, Wiener Lab, Rosario, Argentina).

### 4.7. Histological Analysis

Rat kidney tissues were fixed in formalin, then paraffin embedded, and 5 μm sections were mounted on silanized slides. Immunodetections were performed while using primary anti-αSMA (sc130617) from Santa Cruz Biotechnology, anti-cleaved Caspase-1 (Asp296) (cat nº 673145), and anti-cleaved Caspase 3 (Asp175) (cat nº 96645) from Cell Signaling and anti-NFκB-p65 (ab16502) from Abcam in blocking solution overnight at 4 °C. Immunosignals were visualized while using the LSAB+ System–HRP (DakoCytomation, Carpinteria, CA, USA). Counterstaining were done using Hematoxylin and eosin. The cellular distribution of Caspase-1 in the kidney was established when comparing with the pattern of staining of aquaporin-1 and aquaporin-2, as previously discussed [31]. For immunofluorescent staining, the sections were incubated with anti-NFκB-p65 (ab16502) from Abcam and Alexa Fluor 488-conjugated secondary antibodies (Zhongshan Golden Bridge Biotech, Beijing, China) Nuclei were stained while using DAPI. Images were captured using a fluorescence microscope Leyca DM1000LED. Images were analyzed using the software ImageJ with Color Deconvolution plugin (National Institute of Health, USA).

### 4.8. The isolation of Proximal Tubules from Rats

Renal PCT were extracted from healthy and diabetic rats, as described previously [59]. The kidneys were perfused through the renal artery, first with Hank’s Balanced Salt Solution (HBSS) and then with HBSS containing 1 mg/mL collagenase type II. Renal cortical slices were incubated for 10 min with HBSS/collagenase and maintained at 37 °C, 50 mg/mL BSA was then added, maintaining agitation for another 5 min. The homogenate was poured through the gauze to eliminate non-digested tissue. The extracts were centrifuged at 300× *g* for 3 min. and the pellets were then washed with HBSS without CaCl_2_ and centrifuged. The remaining material was resuspended in 45% Percoll (GE Health Care, Waukesha, WI, USA) in HBSS solution without CaCl_2_ and centrifuged at 7900× *g* for 15 min. at 4 °C, obtaining three phases. The middle phase, containing the PCT was extracted and washed. The pellet was diluted between eight to 14 times, depending on the amount. The PCT extracts were used for western blot assays.

### 4.9. HK2 Cells Treatments

HK2 cells from ATCC (CRL-2190) were serum starved and incubated by 24 h in medium DMEM-F12 containing 5 mM or 25 mM of d-glucose. Subsequently, the cells were treated with the ADORA3 agonist MECA (1 μM) or the ADORA3 antagonist MRS1220 (10 nM) For 24 h in standard culture conditions. The cells were lysed and total protein extracts were used for western blot analysis of cleaved caspase 1 and β-actin.

### 4.10. Western Blots

Protein extracts (50 µg) were fractionated by 10% SDS-PAGE and then transferred to PVDF membranes. The blots were washed with wash buffer (PBS1x, 0.05% Tween20), blocked for 1 h with 0.1% BSA, and then incubated with primary antibodies anti-cleaved Caspase-1 (Asp296) from Cell Signaling and anti-cleaved Caspase 3 (Asp175) (cat nº 96645) from Cell Signaling. The protein levels were expressed as the ratio between the target protein and ß-actin or tubulin detected in the same membrane.

### 4.11. Statistical Analysis

The values are means ± SD, where *n* indicates the number of animals. Comparisons between two and more groups were performed by means of the unpaired Student t test and two-way ANOVA, respectively. If the ANOVA demonstrated a significant interaction between variables, post hoc analyses were performed by the multiple-comparison Bonferroni correction test. *p* < 0.05 was considered to be statistically significant.

## Figures and Tables

**Table 1:** Physiological parameters of experimental groups.

	Non-Diabetic Control Rats	Diabetic Rats	ADORA3 Antagonist-Treated Diabetic Rats
Body Weight (gr)	483 ± 56	257 ± 70 *	192 ± 54 *
Glycaemia (mg/dL)	115 ± 14	542 ± 82 *	522 ± 80 *
Relative kidney weight (g/Kg)	4.76 ± 0.99	9.83 ± 0.63 *	8.44 ± 1.9 *
Proteinuria (mg/mg creatinine)	1.1 ± 0.2	5.0 ± 0.4 *	1.93 ± 0.3 ^#^

Values are means ± S.D. *, *p* < 0.05 vs. non-diabetic control rats. ^#^, *p* < 0.05 vs. diabetic rats. Non-diabetic control rats *n* = 6, diabetic *n* = 5, ADORA3 antagonist-treated diabetic rats *n* = 6.
